# Leveraging social networks to expand HIV self-testing among culturally diverse MSM in Australia: insights from a CFIR-informed process evaluation

**DOI:** 10.3389/fpubh.2026.1797893

**Published:** 2026-06-01

**Authors:** Ying Zhang, Weiming Tang, Michael Traeger, Judith A. Dean, Eric P. F. Chow, Lei Zhang, Cheryl C. Johnson, Mark Stoové, Jason J. Ong, Tiffany R. Phillips

**Affiliations:** 1 School of Translational Medicine, Faculty of Medicine, Nursing and Health Sciences, Monash University, Melbourne, VIC, Australia; 2 Melbourne Sexual Health Centre, Bayside Health, Melbourne, VIC, Australia; 3 Department of Medicine, University of North Carolina at Chapel Hill, Chapel Hill, NC, United States; 4 Burnet Institute, Melbourne, VIC, Australia; 5 School of Public Health and Preventive Medicine, Monash University, Melbourne, VIC, Australia; 6 School of Public Health, Faculty of Health, Medicine and Behavioural Sciences, The University of Queensland, Brisbane, QLD, Australia; 7 Melbourne School of Population and Global Health, The University of Melbourne, Melbourne, VIC, Australia; 8 Phase I Clinical Trial Research Ward, The Second Affiliated Hospital of Xi’an Jiaotong University, Xi'an, Shaanxi, China; 9 China-Australia Joint Research Center for Infectious Diseases, School of Public Health, Xi’an Jiaotong University Health Science Center, Xi’an, Shaanxi, China; 10 Global HIV, Hepatitis and STI Programmes, World Health Organization, Geneva, Switzerland; 11 Australian Research Centre in Sex, Health and Society, La Trobe University, Melbourne, VIC, Australia; 12 Faculty of Infectious and Tropical Diseases, London School of Hygiene and Tropical Medicine, London, United Kingdom

**Keywords:** consolidated framework for implementation research, HIV self-test, men who have sex with men, qualitative, social network

## Abstract

**Background:**

HIV self-testing (HIVST) can help overcome barriers to clinic-based testing among men who have sex with men (MSM). Peer-led distribution through existing social networks may further extend reach and acceptability.

**Objective:**

Guided by the Consolidated Framework for Implementation Research (CFIR), this study evaluated a social network-based HIVST intervention in Australia, in which “test promoters” distributed HIVST kits to friends and sexual partners, to identify facilitators and barriers to implementation.

**Methods:**

Online semi-structured interviews were conducted with 22 MSM, including both test promoters and recipients involved in social network-based HIVST distribution trial. Interviews explored perceptions of acceptability, feasibility, and contextual appropriateness. Data were thematically analysed using the CFIR framework to identify multilevel implementation determinants.

**Results:**

Participants’ mean age was 33.3 years (SD = 8.2). Three interconnected enablers shaped successful implementation: (1) HIVST as an empowering, low-barrier testing option, enhancing privacy, convenience, and autonomy while mitigating stigma; (2) trust and peer credibility, which normalised test sharing and created culturally resonant pathways; and (3) collective responsibility for health, which sustained engagement and diffusion within MSM networks. HIVST’s privacy, convenience, and autonomy enhanced motivation, while peer reassurance reduced procedural anxiety, particularly around finger-prick sampling among first-time or infrequent testers. However, participants also identified persistent barriers, including culturally rooted discomfort discussing HIV, fear of judgment, and the risk of excluding socially isolated or newly arrived individuals. Social network distribution reframed testing as an act of care and solidarity, mitigating stigma and fostering collective ownership. Participants recommended culturally tailored materials, non-invasive test options, and expanded digital platforms to strengthen inclusion and reach.

**Conclusion:**

Social network-based HIVST represents a feasible, acceptable, and culturally responsive strategy to expand testing among MSM in high-income settings. By leveraging relational trust and community solidarity rather than financial incentives, this model offers a scalable and equitable pathway to advance progress toward national and global HIV prevention targets.

## 1 Introduction

HIV testing is central to achieving the global 95–95-95 targets, providing the essential gateway to both prevention and treatment and enabling early diagnosis to interrupt onward transmission. Routine testing is recommended globally for populations at elevated risk of HIV infection, including men who have sex with men (MSM), with at least annual testing advised and more frequent testing (e.g., every three to six months) recommended for individuals with ongoing risk behaviours (1). Over the past decade, global HIV testing coverage has expanded substantially through the scale-up of community-based services, integration of HIV testing into routine health care, and the introduction of innovative approaches such as HIV self-testing. As a result, an estimated 87% of people living with HIV globally were aware of their HIV status in 2024 (2). Despite this progress, a substantial proportion of infections remain undiagnosed. These gaps are primarily driven by structural, social, and behavioural barriers that limit access to or uptake of testing services, rather than limitations in the accuracy of modern HIV diagnostic technologies (3).

In Australia, an estimated 2,360 people, approximately 8% of those living with HIV, remain undiagnosed (4). Although lower than the global average, this proportion remains a critical public health concern because individuals who are unaware of their HIV status cannot initiate timely treatment and may unknowingly contribute to onward transmission. MSM account for the majority of both new and undiagnosed infections in Australia, underscoring their central role in national prevention efforts (4). National guidelines recommend three-monthly HIV testing for sexually active MSM, however, testing uptake remains below recommended levels (5, 6). Data from the Gay Community Periodic Survey indicate that around 60% of MSM report HIV testing in the previous 12 months, while fewer than one quarter report testing three or more times annually, consistent with guideline recommendations (7). Overseas-born MSM, particularly those from Southeast Asia and Latin America, experience disproportionately high rates of undiagnosed HIV (4). These disparities are shaped by intersecting structural, sociocultural, and logistical barriers including stigma, confidentiality concerns, limited healthcare access, and a lack of culturally responsive services, which reduce engagement with traditional facility-based testing models (3, 8). Although similar inequities have been observed in other high-income settings they appear particularly pronounced in Australia, where migration-related factors and restricted Medicare eligibility further constrain access to testing. Addressing these persistent gaps requires culturally informed, community-led implementation strategies capable of reaching individuals who are underserved by conventional health systems.

Globally, social network-based approaches have emerged as effective strategies for increasing HIV testing, facilitating early diagnosis, and improving linkage to care (9–11). By leveraging peer and community trust, these approaches reach individuals who may be reluctant to engage with formal healthcare systems (12, 13). HIV self-testing (HIVST) enhances these strategies by offering confidential, convenient, and user-controlled testing options that can be distributed through social networks (14). Recognising its potential on testing uptake and linkage to care, WHO recommended the integrating HIVST into national testing strategies in 2016 (15). In Australia, regulatory approval of HIVST kits in 2018 expanded opportunities for decentralised, peer-led models of testing ^6^. However, combining HIVST with social network-based delivery requires sustained investment in community engagement, monitoring, and linkage systems to ensure equitable reach and long-term sustainability.

Despite strong evidence supporting both HIVST and social network-based strategies, successful implementation at scale depends on understanding how these innovations interact with real-world systems, contexts, and users. Implementation science offers a critical framework to explore not only whether interventions work, but how, why, for whom, and under what conditions they can be effectively adopted and maintained (16). The Consolidated Framework for Implementation Research (CFIR) provides a comprehensive structure to guide systematic assessments across domains including intervention characteristics, context, individual influences, and implementation processes (17). Applied across diverse health interventions, CFIR enables identification of multi-level determinants shaping implementation outcomes and informs strategies for adapting and scaling complex innovations.

This study presents a CFIR-informed process evaluation of a national pilot trial implementing social network-based HIVST among MSM in Australia. Using qualitative interviews with participants, we explore contextual, organisational, and interpersonal factors influencing the feasibility, acceptability, and sustainability of this model. By situating findings within an established implementation science framework, this study aims to inform strategies for equitable, community-responsive, and system-integrated expansion of HIVST.

## 2 Methods

This study has been reported in accordance with the Relevance, Appropriateness, Transparency, and Soundness (RATS) guidelines for qualitative research (18).

### 2.1 Study design

This qualitative process evaluation, guided by the CFIR version 2.0, was embedded within a national pilot trial assessing the feasibility and acceptability of a social network-based HIVST intervention among MSM in Australia (17, 19). Qualitative interviews were conducted with a subset of trial participants to explore experiences with the intervention and contextual factors shaping its implementation. The pilot trial recruited participants across multiple Australian states through online platforms, community organisations, universities, and sexual health services.

In the pilot, 100 MSM (both Australian-born and overseas-born) were recruited to act as ‘test promoters’, who distributed HIVST kits within their social networks. Each promoter received four individually packaged HIVST kits by mail (Figure S1), each containing the manufacturer’s self-test device, standard instructional materials, and a trial-developed information booklet providing study instructions and frequently asked questions. Additional items included a condom and a lollipop. The booklet also included guidance on interpreting test results and instructions to seek confirmatory testing at a sexual health clinic or healthcare provider in the event of a reactive result, with links to local sexual health services and online resources for follow-up care. Promoters were encouraged to use one test themselves and distribute the remaining three to friends or sexual partners (referred to as ‘recipients’) within three months. Test promoters were instructed to share the kits with individuals who were preferably overseas-born MSM. Each kit was labelled with a unique results-tracking number to enable monitoring through the post-HIVST survey. All participants were invited to complete an online post-HIVST follow-up survey. Full details of the national pilot intervention trial have been reported elsewhere (19).

### 2.2 Recruitment

Participants for this qualitative study were purposively sampled from the pilot HIVST intervention cohort at the end of the three-month follow-up period. The sample included both test promoters and their social contacts (recipients), spanning a range of ages and HIV statuses (Supplementary Table S1).

In the post-HIVST survey, all trial participants were invited to indicate their interest in participating in an interview. Of the 60 participants who expressed interest, 54 reported that they had never previously used HIVST. Thirty participants returned written consent forms, and 22 ultimately completed interviews.

Those who expressed interest were sent a plain-language statement, provided written informed consent via email, and scheduled a virtual interview.

### 2.3 Data collection

Data were collected through semi-structured interviews guided by the CFIR. The interview guide was developed using CFIR as the primary theoretical framework, which provides a structured approach to identifying determinants of implementation across five domains: intervention characteristics, inner and outer settings, individual experiences, and implementation processes. Using CFIR enabled systematic exploration of contextual, interpersonal, and individual factors influencing the acceptability, feasibility, and implementation of the social network-based HIVST intervention.

The initial interview guide was informed by CFIR constructs and adapted to the context of social network-based HIVST distribution among MSM. Questions explored participants’ demographic characteristics, prior knowledge and experiences with HIV testing, perceptions of HIV self-testing (including accuracy and acceptability), experiences with the intervention (such as kit use, distribution strategies, and privacy concerns), as well as broader social and cultural dynamics influencing implementation and suggestions for improvement (Supplementary Table S2).

The guide was developed collaboratively by the research team: TRP (PhD), an experienced female sexual health researcher; JJO (FAChSHM, PhD), a male sexual health physician specialising in underserved populations; and YZ, a female PhD candidate with experience working with culturally diverse MSM. The team’s diverse clinical, sexual, and cultural backgrounds informed the framing and interpretation of questions. To ensure cultural and linguistic appropriateness, the guide was reviewed by MSM community advisors, including overseas-born members of the target population, whose feedback informed the wording and relevance of questions. Minor refinements were made following early interviews to improve clarity and flow.

Reflexivity was considered throughout the research process. YZ had no prior personal relationships with participants, and participants were informed of her role as a researcher before the interviews. Familiarity with sexual health topics may have facilitated open discussion, while regular analytic discussions within the research team helped reflect on potential assumptions and minimise interpretive bias during coding and analysis.

Semi-structured interviews were conducted virtually between July and August 2025, each lasting 45–60 min. Interviews were held via Zoom using individualised passwords and were digitally recorded with participants’ permission. Participants received a plain-language statement and provided written informed consent via email prior to participation, including consent for video recording. At the start of each interview, participants were reminded of the recording and given the opportunity to decline or withdraw. Camera could be disabled to enhance comfort and privacy. Upon completion, participants received an AUD50 (USD33) gift card as remuneration. Immediately after each interview, contextual notes on non-verbal cues and interview dynamics were recorded as field notes. Audio recordings were professionally transcribed verbatim by an independent transcription service. All identifying information was removed, and the transcripts were reviewed by YZ for accuracy.

### 2.4 Data analysis

Interview transcripts were imported into NVivo 15 (QSR International) for coding and thematic analysis. We used a deductive-inductive approach guided by the CFIR framework. CFIR was applied as a sensitising framework rather than a rigid coding template. Transcripts were first deductively coded to preselected CFIR constructs spanning the five domains (Intervention Characteristics, Outer Setting, Inner Setting, Characteristics of Individuals, and Process). We did not deductively code to all CFIR constructs within each domain. Instead, constructs most relevant to the implementation of social network-based HIVST were prospectively selected to preserve analytic depth and avoid forcing data into weakly aligned categories. These included relative advantage, design quality and packaging, complexity, evidence strength and quality, stigma and cultural context, relational connections, implementation climate, capability, motivation, engaging, and reflecting and evaluating. Inductive analysis was then used to identify patterns and generate illustrative examples within each construct. The relationship between CFIR constructs, coding categories, and emergent themes is presented in Supplementary Table S3.

Coding followed a consensual qualitative research approach (20). YZ conducted initial coding and thematic categorisation, with periodic validation by TRP on a subset of transcripts. Regular analytic meetings were held to refine codes, resolve discrepancies, and maintain thematic consistency. A coding framework was developed iteratively and stabilised as analysis progressed (21, 22).

Data collection and analysis occurred concurrently. Saturation was assessed through ongoing review of code development and thematic redundancy. By the 22nd interview, no substantially new codes or themes were identified, and the codebook remained stable across subsequent transcripts, indicating that thematic saturation had been reached for the study population. Because interviews included both test promoters and recipients, we monitored whether new insights emerged across both groups during analysis; however, themes were largely consistent across participant roles.

This collaborative approach was used to develop a nuanced interpretation of the data and reduce bias in the data interpretation (23). A descriptive analysis of the demographic data was conducted using Stata 17 (StataCorp LP, College Station, USA). We report outcomes using the COREQ guidance for qualitative research interviews (Supplementary Table S4).

### 2.5 Data trustworthiness

To ensure data trustworthiness, credibility, dependability, transferability, and confirmability were employed (24). Credibility was established through iterative questioning, member checking (participants reviewed interview transcripts), and established qualitative methods (25). The participants were provided with a copy of the study transcript for checking within a week of completing their interview to minimise recall bias. Twenty participants responded with no changes, except two who replied with minor changes to their transcripts adding clarity to what they already said. Dependability was ensured through detailed methodological descriptions. Transferability was enhanced by providing extensive methodological details (25). Confirmability was supported by including verbatim participant quotes (25).

### 2.6 Ethical considerations

Ethical approval was obtained from the Alfred Hospital Human Research Ethics Committee, Melbourne, Australia (Project number: 322/24). The study was registered with the Australian New Zealand Clinical Trials Registry (ACTRN12625000342415). Participants provided informed consent before participation. Data confidentiality and participant privacy were rigorously maintained throughout the study.

## 3 Results

A total of 22 MSM participants were interviewed, comprising both test promoters and recipients (Table 1). The mean age was 33.3 years (SD = 8.2). Perspectives from both groups are presented across themes, with distinctions noted where experiences or perceptions diverged. One test promoter reported living with HIV prior to participation and used the HIVST to assess its performance. As expected, the test returned a reactive result. Because the participant was already aware of their HIV status, this experience did not materially influence the themes identified across the dataset.

**Table 1:** Participant demographics (*N* = 22).

Participant characteristic	Test promoters (%) *N* = 12*	Social contacts (%) *N* = 11*
Age (years)		
18–24	1 (8%)	2 (18%)
25–34	5 (42%)	7 (64%)
≥35	6 (50%)	2 (18%)
Sexual identity		
Gay	9 (75%)	9 (82%)
Bisexual	2 (17%)	2 (18%)
Other (Pansexual)	1 (8%)	0 (0%)
HIV status		
Positive	1 (8%)	0 (0%)
Negative	11 (92%)	11 (100%)
Country of origin		
Australia	2 (17%)	1 (9%)
China	4 (33%)	2 (18%)
Colombia	1 (8%)	0 (0%)
India	1 (8%)	1 (9%)
Indonesia	1 (8%)	1 (9%)
Italy	0 (0%)	1 (9%)
Malaysia	1 (8%)	1 (9%)
Philippines	1 (8%)	2 (18%)
Singapore	1 (8%)	0 (0%)
Thailand	0 (0%)	1 (9%)
Vietnam	0 (0%)	1 (9%)
Length of time in Australia		
≤ 12 months	1 (8%)	3 (27%)
1–5 years	4 (33%)	4 (36%)
6–10 years	3 (25%)	4 (36%)
>10 years	4 (33%)	0 (0%)
Ever used HIVST prior to trial participation		
No	12 (100%)	10 (91%)
Yes	0 (0%)	1 (9%)
Residential location in Australia by state		
Victoria	11 (92%)	10 (91%)
Other Australian states	1 (8%)	1 (9%)
Education level completed		
Diploma	0 (0%)	1 (9%)
Bachelor	8 (67%)	6 (55%)
Master/postgraduate diploma	4 (33%)	3 (27%)
PhD	0 (0%)	1 (9%)
Visa and residency status		
Student	3 (25%)	3 (27%)
Work/temporary graduate visa	5 (41%)	5 (45%)
Partner/spousal	0 (0%)	1 (9%)
Permanent residency	2 (17%)	1 (9%)
Citizen	2 (17%)	1 (9%)

HIV, human immunodeficiency virus; HIVST, HIV self-testing; PhD, Doctor of Philosophy.

*Numbers do not sum to 22 because one participant initially participated as a social contact and subsequently enrolled as a test promoter.

Guided by the CFIR, the analysis identified five interrelated domains shaping the implementation and experience of the social network-based HIVST intervention: intervention characteristics, outer setting, inner setting (relational context within peer networks), characteristics of individuals, and implementation processes. Together, these domains illustrate how contextual, interpersonal, and individual factors interacted to influence feasibility and acceptability of the intervention.

**Figure 1:**
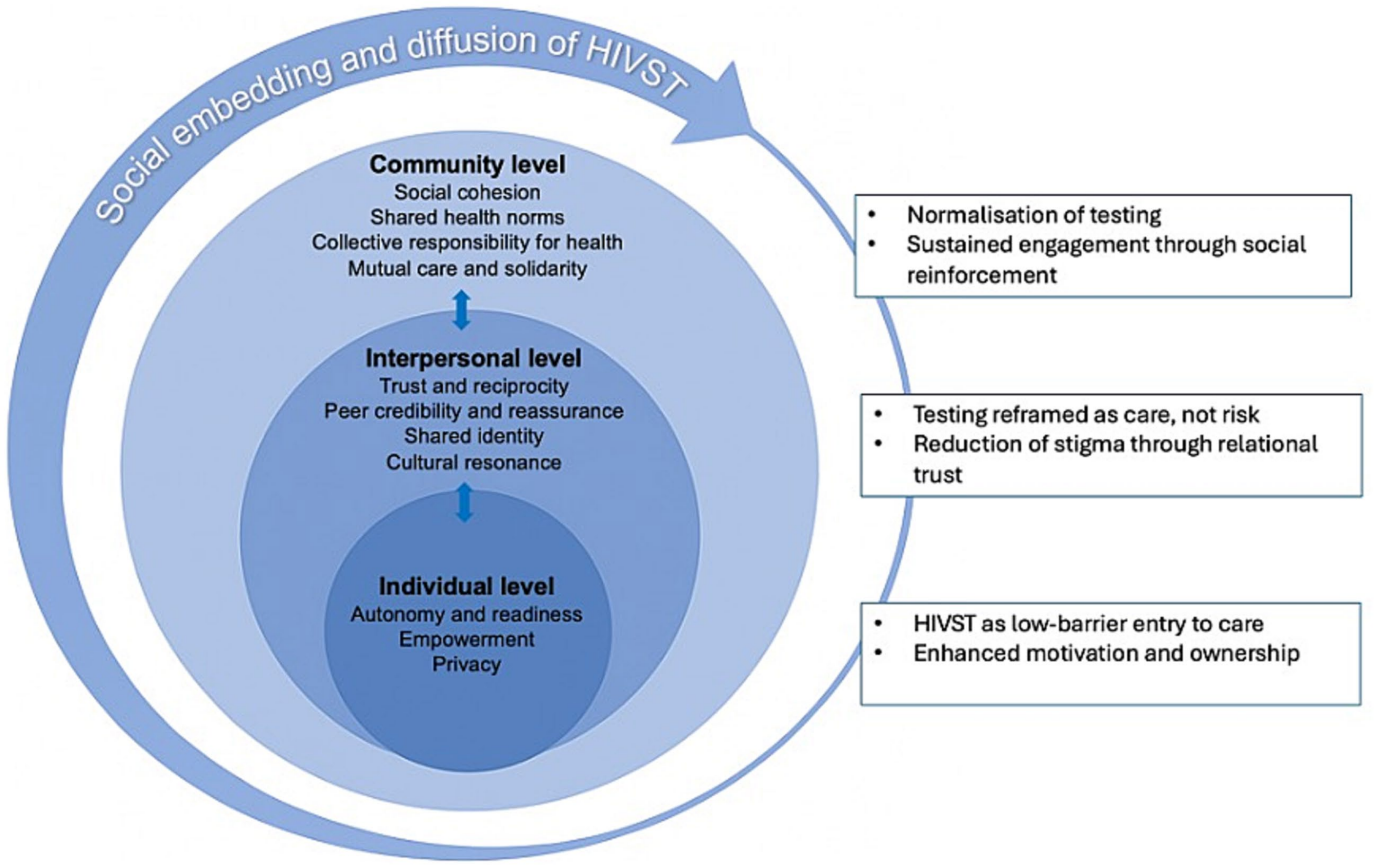
Conceptual framework illustrating mechanisms of social embedding and diffusion of HIVST among MSM.

Analysis across these domains identified three interconnected mechanisms that appeared to shape implementation (Figure 1). First, HIVST functioned as an empowering, low-barrier entry point to care, enhancing privacy, convenience, and autonomy while reducing stigma associated with facility-based testing. Second, trust and peer credibility facilitated uptake by normalising test sharing and creating culturally resonant, socially acceptable pathways to testing. Third, a sense of collective responsibility for health sustained engagement and diffusion within MSM networks, reinforcing norms of mutual care and reciprocity. The conceptual framework presented in Figure 1 represents an interpretive synthesis of themes derived from the CFIR-guided analysis and participant narratives rather than a formal causal model.

### 3.1 Intervention characteristics

#### 3.1.1 Relative advantage

Both test promoters and recipients perceived HIVST as more accessible and acceptable than traditional facility-based serological testing. The flexibility, convenience, and privacy of home-based testing were key advantages, particularly for individuals with busy schedules, limited access to clinics, or discomfort in clinical environments.

*“It takes a lot of effort to go to the clinic—booking, waiting, travelling. But with the kit, I could just do it at home when it suited me.” (P18, social contact, 24 years old).*

Participants also valued the autonomy HIVST provided, describing it as a user-friendly and empowering approach that promoted personal responsibility for health.

#### 3.1.2 Design quality and packaging

Both groups appreciated the discreet, professional, and thoughtfully presented design of the HIVST packages. Promoters described the external packaging as non-identifiable, reducing concerns about stigma when mailing or distributing kits, while recipients valued this privacy during delivery and use.

*“The package is very discreet… No one could guess what it was unless they opened it. That helps with the privacy.” (P01, test promoter, 36 years old).*

#### 3.1.3 Complexity

Overall, participants found the HIVST process straightforward. Among first-time users, however, two types of initial hesitation were common. For some, the finger-prick raised concerns about anticipated pain or general needle phobia. Others worried more about ‘doing it wrong’, reflecting uncertainty about performing the test correctly. Although different in nature, both types of apprehension contributed to perceptions of the process as potentially complex before use. Test promoters addressed these concerns through reassurance and shared experience, sometimes guiding peers in person or remotely. As one test promoter explained,

*“I thought pricking the finger would be quite painful, but actually I did not feel anything… A lot of people are scared of needles. But I told them [friends], as somebody like me who is also scared of needles, I do not even feel it.” (P05, test promoter, 31 years old).*

Instructional materials, including the trial-developed pictorial booklet and manufacturer’s video guide, were especially valued, particularly among first-time testers or those with limited English proficiency, who reported that these visual tools reduced anxiety and supported confidence.

*“It was very helpful, the instructions and the diagrams, and I like that it came with pictures. The instructions were very clear to understand… Even myself I’m not an English speaker, I could understand it properly with no difficulty at all.” (P06, test promoter, 39 years old).*

#### 3.1.4 Evidence strength and quality

Participants expressed high confidence in the accuracy and reliability of the HIVST, citing approval by the Therapeutic Goods Administration (TGA) and distribution through a reputable research institution as key reassurances. For test promoters, referencing this endorsement helped strengthen credibility when introducing kits to peers. Several participants noted that their trust was reinforced by familiarity with the testing technology, such as its similarity to rapid tests used in clinics or COVID-19 diagnostics. The perception that HIVST was supported by scientific evidence and institutional endorsement helped build legitimacy and reduce doubts.

*“It was approved by the TGA, then I’m comfortable that it would have passed through certain high standards… It’s similar to COVID self-test except that you need to prick your finger…” (P10, test promoter, 58 years old).*

Participants understood the need for confirmatory testing following reactive results, viewing HIVST as a complementary approach that expanded choice without replacing clinical services.

*“It’s like a pregnancy test; you do it at home, and if you really want to make sure you are positive, you go to the clinic for the blood test” (P03 test promoter, 23 years old).*

### 3.2 Outer setting

#### 3.2.1 Stigma and cultural context

Both promoters and recipients identified stigma, particularly fears of being seen or judged at clinics, as a major barrier to facility-based testing. These concerns reflected HIV- and sexuality-related stigma and cultural expectations around privacy, discretion, and maintaining social reputation. HIVST mitigated these concerns by offering a discreet and autonomous alternative.

*“At the clinic, you worry who might see you and what they think. But doing it at home, with a kit from a friend, it just felt normal.” (P13, social contact and test promoter, 33 years old).*

*“There’s always that little embarrassment of walking into the clinic. The self-test made it easier. I could just do it when I felt ready.” (P04, social contact, 29 years old).*

The discretion of self-testing was highly valued, allowing users to avoid unwanted disclosure. Test promoters also found that this privacy made it easier to introduce kits within their social circles, framing testing as an act of mutual care rather than a clinical or stigmatised activity.

*“You walk into a clinic, and you are already worried who might see you. But with this, no one needs to know unless you want them to.” (P03, test promoter, 23 years old).*

Self-testing was described as empowering and approachable, particularly for those who might otherwise avoid or delay testing. While participants recognised that confirmatory testing or PrEP initiation required clinical engagement, HIVST served as a psychologically safe first step, offering control and privacy while enabling users to seek further care when ready.

Social network distribution resonated strongly with culturally influenced preferences for indirect communication and relational trust. For some, especially those from Asian and Latin American backgrounds where discretion and reputation were highly valued, receiving a kit from a friend provided a non-confrontational, socially acceptable way to engage in testing.

*“I knew he was doing it too, so it did not feel like something shameful… In my culture, we do not talk about this openly, so it was good to get it from a friend without making it awkward.” (P08, social contact, 32 years old).*

Social network involvement helped reframe HIV testing from a stigmatised medical act to a gesture of care and solidarity by embedding it within trusted, culturally resonant relationships. This shift was most evident among participants with close, supportive ties within MSM or migrant peer networks, where mutual aid and discreet communication about sexual health were already normative.

### 3.3 Inner setting: relational context within peer networks

#### 3.3.1 Relational connections

Most test promoters were hesitant to offer HIVST kits to casual sexual partners (defined as non-regular or short-term sexual contacts) due to limited trust and fears of being misunderstood. Timing also influenced comfort: offering before sex risked seeming distrustful, while offering after sex could imply regret.

*“My main concern was not seeming like I was offering them the test because we’d just done something risky or somehow giving the impression that maybe I was HIV positive and we were testing for whether I transmitted it.” (P02, test promoter, 33 years old).*

Nonetheless, a small subset did offer kits to casual hookups when the opportunity arose. These encounters were often perceived as low-stakes opportunities to introduce the kits, given the lack of long-term relational investment. Some participants expressed confidence that even within casual contexts, such conversations could be handled with maturity and openness.

*“I think that most people who have just had an intimate moment together would be mature enough to be able to handle that.” (P01, test promoter, 36 years old).*

Conversely, established friendships and shared cultural or sexual identities provided a trusted foundation for offering and accepting kits, facilitating open and non-judgmental dialogue.

*“I trust the person who’s giving it to me, and I know that he’s doing it because he’s not judging me but rather because he’s concerned and he wants to help me.” (P07, social contact, 25 years old).*

Among overseas-born MSM, close-knit cultural networks enhanced confidentiality and mutual understanding, making peer-led HIVST particularly effective. Some participants with healthcare backgrounds reported that their professional identity increased credibility (i.e., legitimise the conversation) without undermining health services, viewed instead as an extension of community-based health promotion.

*“As a healthcare professional, people trust me; it’s easier to distribute because they see me as knowledgeable.” (P06, test promoter, 39 years old).*

#### 3.3.2 Implementation climate

Participants described a supportive social climate for peer-led HIV testing, grounded in shared values of health promotion and mutual responsibility, and community care. The intervention was perceived to align with MSM community norms of “looking out for each other,” making social network-distribution a natural extension of existing social support systems.

*“Giving a test to a friend feels natural. It’s how we look out for each other in our community… I think it’s a snowball effect. I received it from my friend, and then I passed it to my other friends, and then my other friends will do the same to their friends.” (P09, test promoter, 36 years old).*

This sense of collective ownership fostered a ripple effect: recipients often became promoters, amplifying reach and reinforcing community ownership of testing.

*“It’s actually putting people together. It’s a very good thing. When you pass on to your peer, you are also sharing information with them, and they go on to share with their friends. It becomes like a chain reaction.” (P21, test promoter, 28 years old).*

### 3.4 Characteristics of individuals

#### 3.4.1 Capability

Confidence in using and promoting HIVST was high, especially among those with prior HIVST experience or healthcare backgrounds. Many felt capable of supporting peers through the process, offering guidance and even co-testing if needed. Younger MSM, in particular, expressed enthusiasm for offering such support, often describing HIVST as a convenient and approachable way to engage friends in testing.

*“My friends trusted me and I shared my experience… After trying it myself, I felt confident showing my friends how to use it too. I am happy to do the test with them if they need my help.” (P05, test promoter, 31 years old).*

Self-efficacy extended beyond technical ability to include interpersonal competence, such as navigating sensitive conversations and offering reassurance.

*“In my culture, HIV is still very stigmatised, and we do not really talk about sex openly. But when my cousin came over, I used the opportunity to give him the kit and also have a sex talk with him. It wasn’t just about the test; it was about making him feel safe and informed.” (P11, test promoter, 28 years old).*

#### 3.4.2 Motivation

Motivation extended beyond convenience or incentives. Participants were driven by altruism, a sense of social responsibility, and the desire to reduce stigma and increase testing rates. Many saw themselves as part of a broader public health effort.

*“I saw it as a way to take responsibility, not just for myself, but for my community. Although it’s not a replacement for clinic testing, it’s a good first step, especially for people who might not test otherwise.” (P12, test promoter, 39 years old).*

Social network distribution was perceived as non-judgmental and supportive, making recipients feel safer and more understood.

*“I think it helps with minimising the stigma… knowing my friend is doing the test too made it easier for me.” (P14, social contact, 40 years old).*

Incentives were appreciated as practical acknowledgements rather than primary motivators.

*“The small incentive was a nice bonus and made it feel worthwhile.” (P02, test promoter, 33 years old).*

### 3.5 Implementation process

#### 3.5.1 Engaging

Peer trust was identified as a key facilitator for offering kits. Recipients were more receptive when approached by trusted peers who shared cultural or sexual identities. Participants felt that receiving test kits from friends normalised the testing process, making the offer feel non-clinical and well-intentioned.

*“I took the kit because it came from a friend I trust, someone who understands me and where I come from. It did not feel like a medical thing; it felt like something we do to look out for each other. My friend was doing it, so I felt comfortable trying it myself.” (P08, social contact, 32 years old).*

Test promoters also adapted their outreach strategies to the recipient’s comfort levels and background, demonstrating cultural and interpersonal sensitivity.

*“I crafted each message differently… based on their background and personality.” (P10, test promoter, 58 years old).*

Some recipients became new distributors, illustrating the intervention’s ability to mobilise community advocates and sustain expansion.

*“My friend gave it to me, and then I passed it on to someone else. It felt good to be part of that.” (P13, social contact and test promoter, 33 years old).*

#### 3.5.2 Reflecting and evaluating

Participants provided constructive feedback to strengthen inclusivity and reach. While focusing on overseas-born MSM was understood, some felt this emphasis excluded other groups who could benefit.

*“It felt restrictive to only target overseas-born individuals. Some local-born friends who could benefit from this missed out.” (P15, social contact, 27 years old).*

Social networks often mirrored cultural or linguistic ties, which shaped distribution patterns. However, cross-group diffusion also occurred as kits flowed between migrant and local-born MSM.

Participants recommended expanding reach through more inclusive messaging, broader public campaigns, and culturally tailored approaches (e.g., multilingual instructions, community ambassadors, visibility at pride or sports events). They also suggested usability refinements: offering a choice of test modality (blood-based or, where feasible, oral-fluid) and clearer post-result pathways, including confirmatory testing for reactive results.

*“Use TikTok, dating apps, posters in public areas… promote through gay sports groups… even booths at pride events.” (P20, social contact, 39 years old).*

## 4 Discussion

This study found that the successful implementation of social network-based HIVST among MSM in Australia was driven by three interconnected enablers: (1) HIVST as an empowering, low-barrier testing option, which enhanced privacy, convenience, and autonomy while reducing stigma; (2) trust and peer credibility, which normalised test sharing and created culturally resonant, socially acceptable pathways to testing; and (3) collective responsibility for health, which sustained engagement and diffusion within MSM networks. Guided by the CFIR, these factors operated across individual (autonomy and readiness), interpersonal (trust and reciprocity), and community (social cohesion and shared norms) levels to create a socially embedded environment where HIVST circulated organically through existing relationships. Rather than representing discrete CFIR constructs, these mechanisms synthesise relationships across multiple CFIR domains and constructs, illustrating how implementation determinants interact within social networks rather than operating as isolated factors. This relational ecosystem transformed HIV testing from a private or clinical act into a shared practice of care and solidarity. The findings align with “pay-it-forward” and other peer-led models that situate public health action within networks of mutual support (13, 26). Extending such strategies to more fragmented or less cohesive communities may require early investment in trust-building, peer leadership development, and culturally resonant communication to reproduce the connective mechanisms that underpin successful diffusion within MSM networks.

Building on these insights, this study proposes a practical framework for social embedding and diffusion that links individual empowerment with network-level trust and community reciprocity. This framework highlights the social conditions that enable innovations such as HIVST to diffuse organically. Applying it beyond MSM populations requires first assessing the strength of local social ties, peer trust, and cultural norms surrounding health and privacy. In settings where networks are weaker or less cohesive, early engagement should prioritise strengthening relational trust and community identity before distribution begins. Mapping these dynamics during planning can help identify leverage points for engagement and guide the design of interventions that are socially grounded, context-sensitive, and sustainable.

At the individual level, HIVST’s privacy, flexibility, and autonomy enhanced motivation and readiness to test, overcoming logistical and psychosocial barriers associated with clinic-based testing. These benefits were amplified by network-based distribution, which allowed discreet access through trusted peers. Consistent with evidence from the United States (27, 28), South Africa (29, 30), and other contexts, peer distribution extended reach to individuals underserved by conventional health services. Discreet packaging and intuitive design further reinforced confidence and reduced stigma (15). While HIVST was generally viewed as straightforward, peer reassurance was particularly valuable for first-time or infrequent testers who anticipated pain or procedural difficulty (31, 32). As self-testing technologies evolve, newer urine- and oral-fluid–based kits showing increasing uptake in China may further enhance acceptability by offering non-invasive alternatives (33).

At the interpersonal level, trust and reciprocity were central to overcoming stigma and fostering participation. Although Australia’s sexual health environment is generally supportive, overseas-born MSM, particularly those from Asian and Latin American backgrounds, described culturally rooted discomfort with HIV-related activities and fear of judgement. These experiences align with evidence that migrant communities face layered vulnerabilities shaped by cultural expectations, stigma, and limited engagement with mainstream services (28, 34). Within these contexts, social network distribution reframed testing as an act of care and solidarity rather than a marker of risk. Peer reassurance, co-testing, and shared participation normalised HIVST, reduced emotional barriers, and aligned testing with cultural expectations of discretion and mutual support. These findings echo evidence from United Kingdom, South Africa, and Kenya, showing that embedding testing in trusted relationships reduces stigma and fosters collective ownership of prevention (13, 26, 35, 36).

At the community level, collective responsibility and social connectivity reinforced sustained engagement and diffusion. Participants described test sharing as a gesture of solidarity that strengthened relational bonds, a dynamic similarly observed in peer-led interventions among transgender women, fishing communities, and MSM networks in Africa (37–39). Embedding HIVST within community relationships thus normalised testing as part of mutual care and wellbeing. However, the same relational dynamics that enabled diffusion also raised equity concerns. Network homophily, the tendency for peers to share demographic, social, or cultural similarities, can shape how health innovations circulate through communities. In network-based distribution models, this may unintentionally reinforce existing social stratification within MSM networks, privileging individuals who are already socially connected while excluding those who are newly arrived, socially isolated, undocumented, or less integrated into community networks. Some test promoters also perceived the focus on overseas-born MSM as potentially restrictive, suggesting that targeted eligibility criteria could limit organic diffusion through trusted relationships. These findings highlight the importance of considering equity in the design of peer-distribution strategies. While social networks can facilitate rapid diffusion of innovations, they may not reliably reach individuals who are marginalised or disconnected from existing community structures. Hybrid delivery models that combine peer distribution with clinic-based services, digital platforms, and community outreach may therefore be necessary to ensure equitable access. Such approaches could provide multiple entry points to HIV testing, allowing individuals to engage through trusted peers while maintaining alternative pathways for those who are less socially connected.

Participants also proposed practical strategies to enhance scalability and integration. These included expanding outreach through social media and community events, improving cultural tailoring with multilingual and inclusive resources, and integrating HIVST with STI and PrEP services framed as supportive rather than diagnostic. Structural adaptations such as discreet pick-up points, vending machines, and simplified reordering systems could further enhance accessibility and preserve privacy. Supporting test promoters through micro-learning resources or light-touch digital training could strengthen capability while maintaining the informal, community-led nature of engagement.

Beyond programmatic design, feasibility and sustainability are central to national adoption. Findings from this study, together with prior reviews, suggest that social motivation and community solidarity may sustain participation in social network-based HIVST distribution even in the absence of financial incentives (13). Participants frequently described altruism, belonging, and collective care as key motivators, while incentives were viewed primarily as acknowledgments rather than primary drivers of engagement. However, these observations reflect qualitative perceptions of acceptability rather than evidence of economic sustainability. Future implementation may therefore benefit from strengthening peer trust, visibility, and recognition alongside existing community networks. Integration with existing established sexual health and PrEP services may further enhance reach and accessibility. However, the economic feasibility of these approaches was not evaluated in this study. Future research should assess cost-effectiveness and scalability, including hybrid delivery models that combine online ordering, pharmacy access, and peer distribution to maximise equity and sustainability within national frameworks.

This study has several limitations. Although the pilot trial recruited participants nationally, the qualitative sample was predominantly based in Victoria reflecting recruitment networks associated with the Melbourne Sexual Health Centre. This geographic clustering may limit the transferability of findings to other Australian jurisdictions. While many findings may be relevant to similar urban sexual health settings, implementation dynamics may differ in regions with different healthcare systems, service availability, or community networks. Although one participant living with HIV used the self-test and received a reactive result as expected, the study did not capture experiences of individuals receiving an unexpected reactive result. Consequently, insights into emotional responses and linkage to confirmatory care following a reactive self-test remain limited. Participation in the qualitative interviews was voluntary and restricted to individuals who completed the post-HIVST survey and expressed interest in being interviewed. This may have introduced self-selection bias, favouring individuals who were more engaged with the intervention, comfortable discussing sexual health, or already connected to MSM peer networks. Younger MSM were underrepresented, and purposive recruitment may have favoured participants already engaged in healthcare, potentially inflating perceptions of acceptability. The sample also consisted largely of participants with higher education levels and stable visa or residency status, which may not reflect the experiences of more marginalised MSM, including those who are socially isolated, undocumented, or less connected to community networks. In addition, a substantial proportion of interview participants were test promoters, whose active role in distributing HIVST kits may have shaped more favourable perceptions of the intervention and limited the range of critical perspectives captured. Social desirability bias may also have influenced responses. As a result, the high levels of acceptability and engagement reported here should be interpreted cautiously.

This qualitative process evaluation examined participants’ experiences and implementation dynamics rather than quantitative outcomes. Measures of testing uptake, confirmatory testing, and linkage to care were assessed in the main pilot trial and reported elsewhere (19). The study also did not evaluate the financial costs or operational burden associated with implementing the intervention, such as kit distribution, logistics, and programme coordination. Future research should assess the economic and implementation requirements of scaling social network-based HIVST models within health systems. Future research should also examine more diverse populations and evaluate downstream outcomes such as confirmatory testing, linkage to care, and PrEP uptake, with particular attention to equity.

## 5 Conclusion

Social network-based HIVST represents a promising and culturally responsive approach to expand testing among MSM in Australia, particularly those born overseas. By harnessing relational trust, peer influence, and clear instructional support, this model enhances acceptability, feasibility, and reach. Strengthening peer leadership and culturally resonant communication will be key to ensuring scalability, equity, and sustained progress toward national and global HIV prevention targets.

## Data Availability

The original contributions generated and analysed during the study are included within the article and its Supplementary Material. Requests to access the datasets should be directed to the corresponding author.
