# Supplementary material for: Leveraging social networks to expand HIV self-testing among culturally diverse MSM in Australia: insights from a CFIR-informed process evaluation
Source: Front Public Health. 2026 Jun 1;14:1797893. doi: 10.3389/fpubh.2026.1797893 (PMC13265509; doi:10.3389/fpubh.2026.1797893)
Supplement: Supplementary file 1 [file Data_Sheet_1.pdf]

Table of Contents

Figure S1. External packaging and contents of each package..2
Table S1. Sampling framework, eligibility criteria, and interview schedule topics............3
Table S2. Interview guide 4
Table S3. Mapping of CFIR constructs to themes identified in the analysis.....................5
Table S4. COREQ (COnsolidated criteria for REporting Qualitative research) Checklist ..6

Figure S1. External packaging and contents of each package

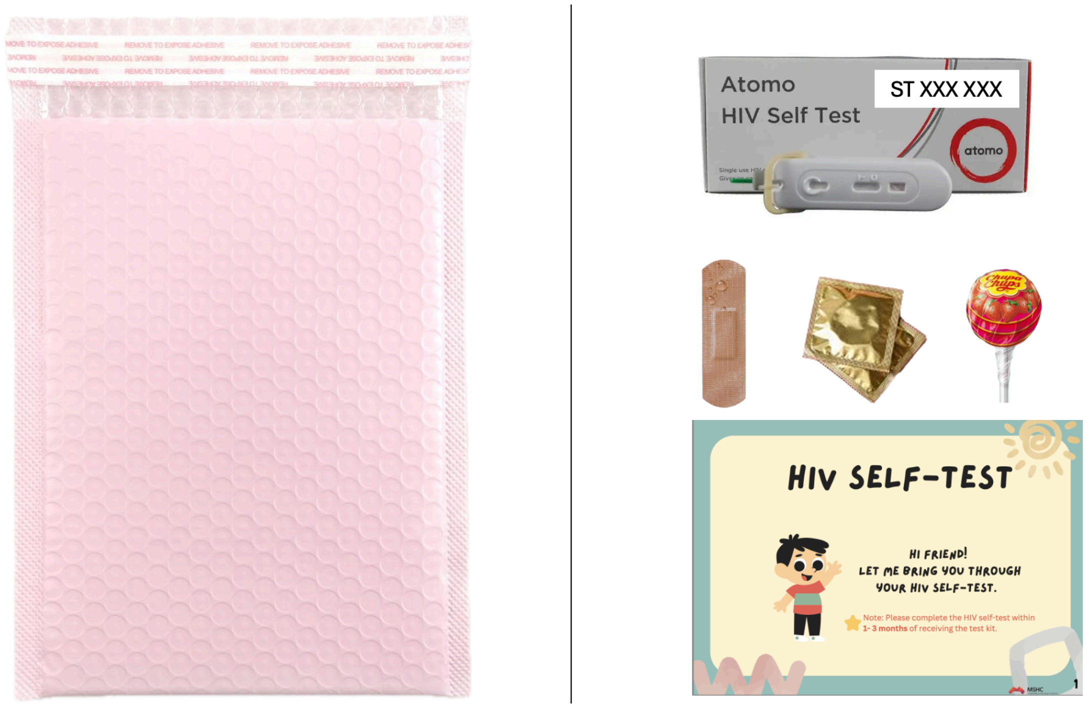

Table S1. Sampling framework, eligibility criteria, and interview schedule topics

Eligibility Criteria	• Gay or bisexual men or other men who have sex with men (MSM)<br>• Cis or transgender men<br>• Aged 18 years or older<br>• All HIV status
Sampling framework	• Range of ages<br>• Test promoter or recipients
Interview schedule topics	• HIV self-test knowledge<br>• Role as test promoter or social contact<br>• Experience in the intervention<br>• Motivation within the study<br>• Willingness to become a test promoter (for recipients)<br>• Suggestions for improvement

HIVST, HIV self-testing; MSM, men who have sex with men

Table S2. Interview guide

Domain	Interview questions
**Participant background**	• Which country were you born in?<br>• What is your nationality?<br>• How old are you?<br>• What is your highest level of education?<br>• What type of visa are you currently holding?<br>• When did you arrive in Australia?<br>• Are you eligible for Medicare?<br>• How would you describe your sexuality?
**Sexual health context**	1. Could you tell me a bit about your current relationship status?<br>2. What is your general approach to HIV prevention in your sex life?
**Prior knowledge and experience with HIVST** *(CFIR: Characteristics of individuals)*	3. Prior to this study, had you ever used an HIVST If yes, could you describe that experience?<br>4. What did you know about HIVST before hearing about this study?
**Experience with the HIVST intervention** *(CFIR: Intervention characteristics)*	5. Can you walk me through your experience with this HIVST program, from hearing about it to using or sharing the kit?
**Intervention design and support materials** *(CFIR: Intervention characteristics – design quality and packaging)*	6. The package you received included an information booklet and a study website with an instructional video. How useful did you find this type of support?
**Perceived reliability and trust in HIVST** *(CFIR: Intervention characteristics – evidence strength and quality)*	7. Did you trust the accuracy and reliability of the HIVST kit? Why or why not?
**Comparison with facility-based testing** *(CFIR: Relative advantage)*	8. Would you prefer self-testing over visiting a traditional healthcare setting? Why?
**Participant role in the program**	9. What did you do with the test you received? (Used it yourself, shared it with others, or both.)
**Experiences receiving HIVST kits** (Recipients only)	10a. How comfortable were you with receiving the self-test kits through this program?<br>10b. Would you feel comfortable giving the self-test kits to your friends or sexual partners?
**Experiences distributing HIVST kits** (Test promoters only)	11a. How comfortable were you with distributing the HIVST kits in this program?<br>11b. How confident did you feel offering or explaining the test to someone else?
**Privacy and confidentiality considerations** *(CFIR: Outer setting / social context)*	12. Were there any concerns about privacy or confidentiality while receiving, using, or sharing the test?
**Barriers and challenges** *(CFIR: Implementation process)*	13. Were there any challenges or awkward moments when using or distributing the test?
**Perceptions of peer-based distribution** *(CFIR: Implementation process – engaging)*	14. What do you think about this peer-based approach to HIV testing overall?
**Recommendations for improvement** *(CFIR: Reflecting and evaluating)*	15. Do you have any ideas or suggestions for how we could improve this program or reach more people?

CFIR, Consolidated Framework for Implementation Research; HIVST, HIV self-testing

Table S3. Mapping of CFIR constructs to themes identified in the analysis

CFIR Domain	CFIR Construct	Example Codes	Emergent Theme
Intervention Characteristics	Relative advantage	Privacy, convenience, autonomy	HIVST as a low-barrier testing option
	Design quality and packaging	Discreet packaging, instructions, usability	Acceptability of HIVST delivery
	Complexity	Finger-prick concerns, need for guidance	Peer reassurance supporting uptake
	Evidence strength and quality	Trust in accuracy, TGA approval, similarity to clinic testing, confidence in technology	Institutional credibility supporting acceptance
Outer Setting	Stigma and cultural context	Fear of judgment, cultural discomfort discussing HIV	Social network distribution reducing stigma
Inner Setting	Relational connections and peer trust	Trust between peers, shared identity	Trust enabling testing conversations
	Implementation climate	Community norms of care	Collective responsibility for health
Characteristics of Individuals	Capability	Confidence explaining test	Peer leadership and support
	Motivation	Altruism, community responsibility	Collective engagement in testing
Implementation Process	Engaging	Peer recruitment strategies	Diffusion through social networks
	Reflecting and evaluating	Participant suggestions	Improving reach and inclusivity

CFIR, Consolidated Framework for Implementation Research; HIVST, HIV self-testing; TGA, Therapeutic Goods Administration

Table S4. COREQ (COnsolidated criteria for REporting Qualitative research) Checklist

COREQ (COnsolidated criteria for REporting Qualitative research) Checklist

A checklist of items that should be included in reports of qualitative research. You must report the page number in your manuscript where you consider each of the items listed in this checklist. If you have not included this information, either revise your manuscript accordingly before submitting or note N/A.

Topic	Item No.	Guide Questions/Description	Reported on Page No.
**Domain 1: Research team and reflexivity**			
Personal characteristics			
Interviewer/facilitator	1	Which author/s conducted the interview or focus group?	8
Credentials	2	What were the researcher's credentials? E.g. PhD, MD	8
Occupation	3	What was their occupation at the time of the study?	7
Gender	4	Was the researcher male or female?	7
Experience and training	5	What experience or training did the researcher have?	8
Relationship with participants			
Relationship established	6	Was a relationship established prior to study commencement?	7
Participant knowledge of the interviewer	7	What did the participants know about the researcher? e.g. personal goals, reasons for doing the research	7
Interviewer characteristics	8	What characteristics were reported about the inter viewer/facilitator? e.g. Bias, assumptions, reasons and interests in the research topic	8
**Domain 2: Study design**			
Theoretical framework			
Methodological orientation and Theory	9	What methodological orientation was stated to underpin the study? e.g. grounded theory, discourse analysis, ethnography, phenomenology, content analysis	8
Participant selection			
Sampling	10	How were participants selected? e.g. purposive, convenience, consecutive, snowball	7
Method of approach	11	How were participants approached? e.g. face-to-face, telephone, mail, email	7
Sample size	12	How many participants were in the study?	7
Non-participation	13	How many people refused to participate or dropped out? Reasons?	7
Setting			
Setting of data collection	14	Where was the data collected? e.g. home, clinic, workplace	7
Presence of non-participants	15	Was anyone else present besides the participants and researchers?	7
Description of sample	16	What are the important characteristics of the sample? e.g. demographic data, date	7
Data collection			
Interview guide	17	Were questions, prompts, guides provided by the authors? Was it pilot tested?	7
Repeat interviews	18	Were repeat inter views carried out? If yes, how many?	NA
Audio/visual recording	19	Did the research use audio or visual recording to collect the data?	7
Field notes	20	Were field notes made during and/or after the inter view or focus group?	7
Duration	21	What was the duration of the inter views or focus group?	8
Data saturation	22	Was data saturation discussed?	8
Transcripts returned	23	Were transcripts returned to participants for comment and/or	8

Topic	Item No.	Guide Questions/Description	Reported on Page No.
		correction?	
**Domain 3: analysis and findings**			
Data analysis			
Number of data coders	24	How many data coders coded the data?	8
Description of the coding tree	25	Did authors provide a description of the coding tree?	8
Derivation of themes	26	Were themes identified in advance or derived from the data?	8
Software	27	What software, if applicable, was used to manage the data?	8
Participant checking	28	Did participants provide feedback on the findings?	8
Reporting			
Quotations presented	29	Were participant quotations presented to illustrate the themes/findings? Was each quotation identified? e.g. participant number	8
Data and findings consistent	30	Was there consistency between the data presented and the findings?	8
Clarity of major themes	31	Were major themes clearly presented in the findings?	8
Clarity of minor themes	32	Is there a description of diverse cases or discussion of minor themes?	8

Developed from: Tong A, Sainsbury P, Craig J. Consolidated criteria for reporting qualitative research (COREQ): a 32-item checklist for interviews and focus groups. *International Journal for Quality in Health Care*. 2007. Volume 19, Number 6: pp. 349 – 357

Once you have completed this checklist, please save a copy and upload it as part of your submission. DO NOT include this checklist as part of the main manuscript document. It must be uploaded as a separate file.
